# Epidemiologic trends and increasing inequalities in orthognathic surgery in Germany: a nationwide analysis 2005–2022

**DOI:** 10.1038/s41598-025-90889-1

**Published:** 2025-02-20

**Authors:** Axel Meisgeier, Simon Pienkohs, Constantin Salomia, Laura Moosdorf, Andreas Neff

**Affiliations:** 1 Department of Oral and Craniomaxillofacial Surgery, UKGM GmbH, University Hospital Marburg and Faculty of Medicine, Philipps University, 35043 Marburg, Germany; 2 Center for Orthopaedics and Trauma Surgery, UKGM GmbH, University Hospital Marburg and Faculty of Medicine, Philipps University, Marburg, Germany

**Keywords:** Epidemiology, Healthcare, Maxillofacial surgery, Orthodontics, Epidemiology, Surgery

## Abstract

Orthognathic surgery (OS) has become a widely used treatment option for moderate to severe dento-skeletal malocclusions. The aim of this study was to analyze the incidence and time trends of OS and potential influencing factors in the German healthcare system. Nationwide data regarding the national diagnosis-related-group (DRG) inpatient billing system was received from the German Federal Statistical Office. We estimated age-gender standardized incidence of OS-associated procedures between 2005 and 2022 and evaluated age- and gender-adjusted time trends using Poisson regression analysis. The total standardized incidence rate of OS-associated procedures in the observational period 2005–2022 was 11.1 (♀13.0; ♂ 9.2) per 100,000 person years. A significant increase of 61.8% (♀+48.7%; ♂+82.9%) in OS-associated procedures was observed within the observational period, mainly due to the increase of the procedures in the age groups 15–34 years (+ 53.6%), 35–59 years (+ 66.7%) and 60–79 years (+ 20.0%). Mandibular procedures increased by 45.0%, while maxillary procedures increased by 88.2%. There were significant differences of the incidence of OS-associated procedures between the various federal states in Germany, indicating a higher incidence and stronger increase in more affluent regions. The age- and gender-standardized incidence of OS-associated procedures increased among all genders and the most relevant age groups in Germany in the last two decades. There is an ongoing trend to implement maxillary and combined procedures. These effects are limited to more affluent regions only, reflecting the impact of socioeconomic and regional factors on healthcare accessibility.

**Supplementary Information:**

The online version contains supplementary material available at 10.1038/s41598-025-90889-1.

## Introduction

Orthognathic surgery has evolved significantly over the past few decades, with advancements reducing morbidity and expanding indications^1^. Concordantly, the field has seen an annual publication growth rate of 9.5% from 1980 to 2022^2^. Orthognathic surgery is a core competence in oral and maxillofacial (OMF) surgery. Patients seeking help for dental and skeletal irregularities and malpositions of the teeth and jaws are an important group in this specialty. The treatment of malpositioned teeth and jaws is based on a combination of maxillofacial surgery and orthodontics and has become well established over the last decades and developed continuously further^3–5^. Jaw deformities are related to a number of aesthetic issues and functional difficulties like chewing problems, swallowing disorders, speech impairment, maxillofacial pain and breathing difficulties^6^. Both aesthetic and functional impairments significantly decrease the quality of life of patients suffering from dentofacial deformities^7,8^. The severity of malocclusion is correlated to the decrease in general self-esteem and dental self-confidence, whereas aesthetic concerns and the psychological and social impact of dental aesthetics increase^9^. While individual patients may vary in their motivations, perceptions and expectations, the primary reasons for seeking treatment are often rooted in a desire to enhance self-confidence, improve orofacial aesthetics, and optimize functional outcomes^10,11^. Recent trends include increased precision through computer-assisted planning, patient-specific fixation and expanded use for upper airway obstruction management^12^. The evolution of orthognathic surgery has been influenced by various factors, including aesthetic motivations, improved orthodontic-surgical collaboration, and the adoption of optimized fixation techniques^13^. Virtual planning and examination of condylar changes post-surgery represent the newest areas of focus in the field^2^. These developments have led to a broader range of applications, including the management of dysmorphosis, obstructive sleep apnea syndrome and facial aesthetics^1^. Three-dimensional planning tools also enable surgeons to provide comprehensive information to patients and referring colleges about the extent of necessary osteotomies and the estimated appearance after the corrective procedures^14,15^.

As it was shown in different single center evaluations that the incidence of orthognathic surgeries is increasing, there are only scarce data on regional or national levels^16–21^. To the best of our knowledge, there is no definitive data from any other Central European country examining epidemiological trends in orthognathic surgery at the national level. The objective of this study was, therefore, to investigate the nationwide incidence of OS-associated procedures in Germany. To this end, we evaluated the overall, regional, gender-specific, and age-related distribution of patients undergoing OS requiring inpatient treatment covered by the German DRG system.

## Materials and methods

The national diagnosis-related groups (DRG) inpatient billing system includes data from all hospitals in Germany that use the DRG system. More than 99% of inpatient treatments are covered. Hospitals are required by law to provide comprehensive information about hospital care, including patient demographics, diagnoses, comorbidities, complications and procedures.

Surgical procedures from the years 2005 to 2022 were coded according to the OPS (Operation and Procedure Classification System), a German modification of the ICPM (International Classification of Medical Procedures). All diagnoses were coded according to the ICD-10GM (German version of the International Classification of Diseases and Related Health Problems, 10th Edition). Detailed lists of all OS-associated procedures per year (coded 5-776 and 5-777 in the OPS) were provided by the German Federal Statistical Office (Statistisches Bundesamt - Destatis. Genesis-Online. Data license by-2-0) and analyzed as already published in a previous study^22^. Due to the retrospective nature of the study with fully anonymous, aggregated data Research Ethics Committee of the Medical Faculty of the Philipps-University-Marburg waived the need of obtaining informed consent (24-351Anz; December 13th, 2024). OS-associated procedures per year (PPY) were calculated and reported. Mean age of patients was calculated. The normality of the distribution of continuous variables was tested by Kolmogorov-Smirnov test. Continuous variables with normal distribution were presented as mean and standard deviation. Means of 2 continuous normally distributed variables were compared by independent samples Student’s t-test. The differences were considered significant at *p* < 0.05. In addition, population-adjusted rates of OS-associated surgeries per 100,000 person years were calculated using population data also provided by the German Federal Statistical Office and reported with 95% confidence interval. In order to avoid seasonal influences, the OS-associated procedures were analyzed on an annual basis. We performed the main analysis for the entire population and the analyses stratified by sex, age group and federal state^22^. We computed crude and age-sex standardized incidence rates of OS-associated procedures for each calendar year, taking the German population of the census 2011 as standard population using 0–14, 15–34, 35–59, 60–79 and ≥ 80 years as age classes to exclude bias due to demographic heterogeneity over time. To ascertain male and female incidence rates, age standardization was performed with the aforementioned age classes using the age distribution of the male and female standard population resulting in different weights for both genders. The calculation of the 95% confidence intervals (95% CI) of crude and standardized incidences was performed by the delta method. Separate Poisson regression models were computed to investigate time trends, fitted with incidence rates of OS-associated procedures as dependent variables and age group, gender, federal state and year of OS-associated procedures starting from baseline year 2005 as independent variables. The age class 35–59 years, female sex was used as reference group. All models were adapted by descale adjustment to account for overdispersion of the outcome variable. In order to evaluate the influence of regional differences, various population-related, economic and health system-related parameters of the individual federal states, provided by the German Federal Statistical Office (Statistisches Bundesamt - Destatis. Genesis-Online. Data license by-2-0). were analyzed in a correlation analysis. Data on the Human development index (HDI) was provided by the United Nations Human Development Program^23^. The correlations were determined using the Pearson correlation coefficient. According to Hemphill et al., there is a weak correlation at *r* < 0.20, a medium correlation at *r* = 0.20 to 0.30 and a strong correlation at *r* > 0.30^24^. The p-value correction for multiple testing was made by adjusting the false discovery rate according to Benjamini & Hochberg^25^. Statistical analysis was performed using IBM SPSS Statistics Version 29.0 (IBM Deutschland GmbH, Böblingen, Germany).

## Results

**Fig. 1:**
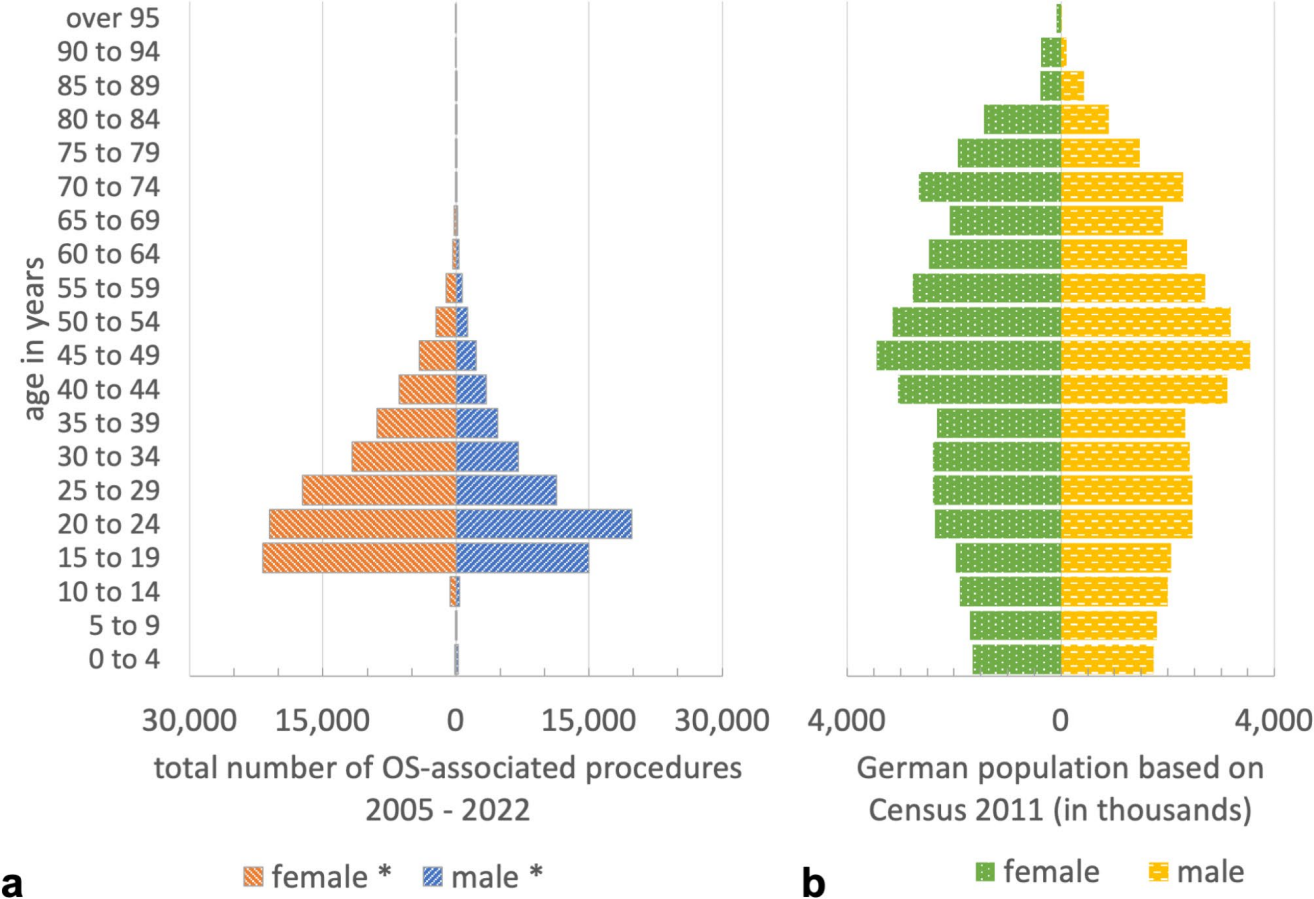
(**A**) Age-gender-diagram of the total number of OS-associated procedures 2005–2022. (**B**) Age-gender-diagram of the German population based on census 2011.

In the observational period 2005–2022 a total of 163,457 OS-associated procedures were registered in Germany and could be included in the study. The distribution regarding age, gender and year of cases treated with OS is presented in Table 1; Fig. 1. The majority of cases were aged 15 years to 34 years (84.9%) and female (58.9%). The mean age within this period increased slightly from 27.3 years between 2005 and 2013 to 27.9 years between 2014 and 2022 (*p* < 0.05).

**Table 1:** Total numbers of OS-associated procedures in Germany 2005–2022.

All OS-associated procedures		Total	Male	Female
Years	Number of procedures Mean age (years, SD)	163,457 27.6 (10.4)	67,145 26.9 (10.3)	96,312 28.1 (10.5)
2005–2022		10,031 27.2 (10.7)	2777 27.6 (10.9)	7254 26.6 (10.5)
2005		9922 27.0 (10.6)	2783 27.4 (10.6)	7139 26.3 (10.5)
2006		10,747 27.1 (10.8)	3052 27.4 (10.8)	7695 26.6 (10.8)
2007		10,938 27.1 (10.7)	3155 27.5 (10.7)	7783 26.6 (10.7)
2008		11,375 27.3 (10.8)	3321 27.7 (10.7)	8054 26.7 (10.9)
2009		11,516 27.3 (10.7)	3280 27.7 (10.9)	8236 26.7 (10.5)
2010		12,533 27.5 (10.9)	3634 27.9 (11.0)	8899 26.9 (10.7)
2011		12,651 27.4 (10.5)	3563 28.0 (10.5)	9088 26.6 (10.6)
2012		12,758 27.7 (10.6)	3673 28.1 (10.4)	9085 27.1 (10.7)
2013		13,065 27.5 (10.4)	3717 28.0 (10.5)	9348 26.7 (10.3)
2014		13,397 27.2 (10.2)	3817 27.7 (10.2)	9580 26.5 (10.1)
2015		13,997 27.4 (10.2)	3994 28.0 (10.4)	10,003 26.4 (9.6)
2016		14,164 28.0 (10.5)	4040 28.8 (10.5)	10,124 26.8 (10.3)
2017		14,514 27.6 (10.1)	4303 28.1 (10.4)	10,211 26.9 (9.6)
2018		14,980 27.9 (10.2)	4482 28.6 (10.4)	10,498 27.0 (9.8)
2019		13,894 28.1 (10.2)	4280 28.5 (10.3)	9614 27.7 (10.0)
2020		14,733 28.2 (10.0)	4549 28.6 (10.0)	10,184 27.6 (10.0)
2022		15,392 29.0 (10.2))	4725 29.6 (10.3)	10,667 28.1 (9.9)
Age				
0–14 years		1665	740	925
15–34 years		125,098	53,304	71,794
35–59 years		35,351	12,454	22,897
60–79 years		1297	627	670
Over 80 years		46	20	26

**Fig. 2:**
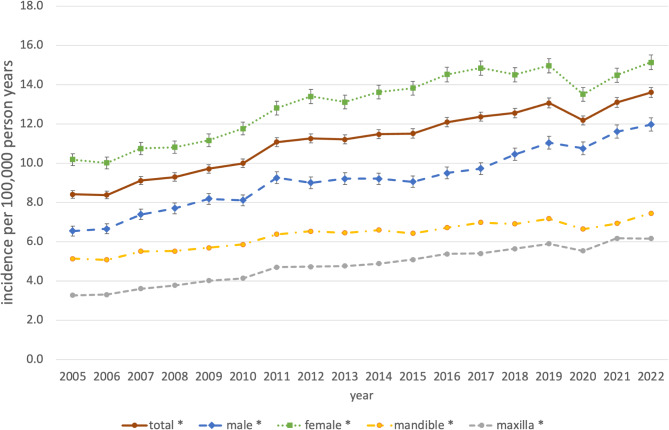
Standardized overall, gender-specific and region-specific incidence of OS-associated procedures, Germany, 2005–2022. *Significant increase (p-value time trend Poisson-Regression model < 0.05).

**Fig. 3:**
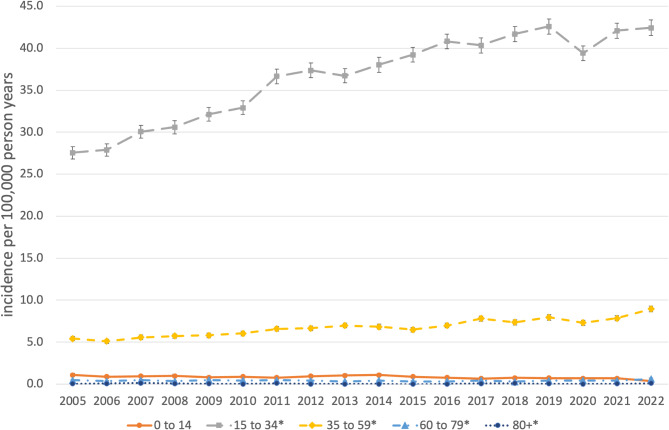
Standardized age-specific incidence of OS-associated procedures, Germany, 2005–2022. *Significant increase (p value time trend Poisson-Regression model < 0.05).

The age and gender standardized incidence rates of OS-associated procedures are shown in Table 2; Figs. 2 and 3 for the total population and stratified by gender and age. The total standardized incidence rate of OS-associated procedures in the observational period 2005–2022 was 11.1 per 100,000 person years [95% CI: 11.1–11.2] ranging from 8.4 [8.2–8.6] in 2005 to 13.6 [13.4–13.9] in 2022, an increase of + 61.8% within the observational period. Gender distribution slightly shifted from 39.1% in males and 60.9% in females in 2005 to 44.2% and 55.8% in 2022, respectively. In males the overall incidence was 9.2 [9.1–9.3] spreading from 6.5 [6.3–6.8] in 2005 to 12.0 [11.6–12.3] in 2022, in females the total incidence rate was 13.0 [12.9–13.1] varying between 10.0 [9.7–10.3] in 2006 and 15.1 [14.8–15.8] in 2022. Among the different age groups adolescents and young adults from 15 to 34 years had the highest total incidence rate with 36.6 [36.4–36.8] differing between 27.6 [26.8–28.3] in 2005 to 42.6 [41.7–43.5] in 2019.

**Table 2:** Incidence of OS associates procedures in Germany 2005–2022. Incidence rates (95% confidence interval) per 100,000 person years standardized to the German population based on the Census 2011.

	Total	Gender		Age group				
		Male	Female	0–14 years	15–34 years	35–59 years	60–79 years	over 80 years
All years	11.1 (11.1–11.2)	9.2 (9.1–9.3)	13.0 (12.9–13.1)	0.8 (0.8–0.9)	36.6 (36.4–36.8)	6.7 (6.7–6.8)	0.4 (0.4–0.4)	0.1 (0.0–0.1)
2005–2013	9.8 (9.8–9.9)	8.0 (7.9–8.1)	11.6 (11.4–11.7)	0.9 (0.9–1.0)	32.4 (32.2–32.7)	6.0 (5.9–6.1)	0.4 (0.4–0.4)	0.1 (0.0–0.1)
2014–2022	12.4 (12.4–12.5)	10.4 (10.3–10.5)	14.4 (14.3–14.5)	0.7 (0.7–0.8)	40.7 (40.4–41.0)	7.5 (7.4–7.6)	0.4 (0.4–0.4)	0.0 (0.0–0.1)
Relative risk	1.27 (1.25–1.28)*	1.30 (1.28–1.32)*	1.24 (1.23–1.26)*	0.80 (0.72–0.88)*	1.26 (1.24–1.27)*	1.25 (1.23–1.28)*	0.98 (0.88–1.10)	0.76 (0.42–1.37)
Year								
2005	8.4 (8.2–8.6)	6.5 (6.3–6.8)	10.2 (9.9–10.5)	1.1 (0.9–1.2)	27.6 (26.8–28.3)	5.4 (5.2–5.7)	0.5 (0.4–0.6)	0.1 (0.0–0.1)
2006	8.4 (8.2–8.6)	6.7 (6.4–6.9)	10.0 (9.7–10.3)	0.9 (0.7–1.0)	27.9 (27.1–28.6)	5.1 (4.9–5.4)	0.3 (0.3–0.4)	0.1 (0.0–0.2)
2007	9.1 (8.9–9.3)	7.4 (7.1–7.7)	10.8 (10.4–11.1)	1.0 (0.8–1.1)	30.1 (29.3–30.8)	5.6 (5.3–5.8)	0.5 (0.3–0.6)	0.1 (0.0–0.2)
2008	9.3 (9.1–9.5)	7.7 (7.4–8.0)	10.8 (10.5–11.1)	1.0 (0.8–1.1)	30.6 (29.8–31.4)	5.7 (5.5–6.0)	0.3 (0.3–0.4)	0.1 (0.0–0.2)
2009	9.7 (9.5–9.9)	8.2 (7.9–8.5)	11.2 (10.8–11.5)	0.8 (0.6–0.9)	32.1 (31.3–32.9)	5.8 (5.5–6.1)	0.5 (0.4–0.6)	0.0 (0.0–0.1)
2010	10.0 (9.8–10.2)	8.1 (7.8–8.4)	11.8 (11.4–12.1)	0.9 (0.7–1.0)	32.9 (32.1–33.7)	6.0 (5.8–6.3)	0.4 (0.3–0.5)	0.0 (0.0–0.1)
2011	11.1 (10.8–11.3)	9.3 (9.0–9.6)	12.8 (12.5–13.2)	0.8 (0.6–0.9)	36.7 (35.8–37.5)	6.6 (6.3–6.9)	0.5 (0.4–0.6)	0.1 (0.0–0.2)
2012	11.3 (11.0–11.5)	9.0 (8.7–9.3)	13.4 (13.0–13.8)	0.9 (0.8–1.1)	37.4 (36.5–38.2)	6.7 (6.4–7.0)	0.4 (0.3–0.5)	0.0 (0.0–0.1)
2013	11.2 (11.0–11.4)	9.2 (8.9–9.5)	13.1 (12.8–13.5)	1.0 (0.8–1.2)	36.7 (35.9–37.6)	7.0 (6.7–7.3)	0.3 (0.2–0.4)	0.0 (0.0–0.1)
2014	11.5 (11.2–11.7)	9.2 (8.9–9.5)	13.6 (13.3–14.0)	1.1 (0.9–1.2)	38.0 (37.1–38.9)	6.8 (6.5–7.1)	0.4 (0.3–0.5)	0.0 (0.0–0.1)
2015	11.5 (11.3–11.7)	9.1 (8.8–9.3)	13.8 (13.5–14.2)	0.9 (0.7–1.0)	39.2 (38.3–40.1)	6.5 (6.2–6.8)	0.3 (0.2–0.4)	0.0 (0.0–0.0)
2016	12.1 (11.9–12.3)	9.5 (9.2–9.8)	14.5 (14.2–14.9)	0.8 (0.6–0.9)	40.8 (39.9–41.7)	7.0 (6.7–7.3)	0.3 (0.2–0.4)	0.0 (0.0–0.1)
2017	12.4 (12.1–12.6)	9.7 (9.4–10.0)	14.8 (14.5–15.2)	0.7 (0.5–0.8)	40.3 (39.4–41.2)	7.8 (7.5–8.1)	0.4 (0.3–0.5)	0.1 (0.0–0.1)
2018	12.6 (12.3–12.8)	10.5 (10.1–10.8)	14.5 (14.2–14.9)	0.7 (0.6–0.9)	41.7 (40.8–42.6)	7.4 (7.1–7.7)	0.3 (0.2–0.4)	0.1 (0.0–0.2)
2019	13.1 (12.8–13.3)	11.0 (10.7–11.4)	15.0 (14.6–15.3)	0.7 (0.6–0.8)	42.6 (41.7–43.5)	7.9 (7.6–8.3)	0.4 (0.3–0.5)	0.0 (0.0–0.1)
2020	12.2 (11.9–12.4)	10.8 (10.4–11.1)	13.5 (13.2–13.9)	0.7 (0.5–0.8)	39.4 (38.5–40.3)	7.3 (7.0–7.6)	0.4 (0.3–0.5)	0.0 (0.0–0.0)
2021	13.1 (12.9–13.3)	11.6 (11.3–11.9)	14.5 (14.1–14.9)	0.7 (0.5–0.8)	42.1 (41.1–43.0)	7.9 (7.5–8.2)	0.4 (0.3–0.5)	0.0 (0.0–0.1)
2022	13.6 (13.4–13.9)	12.0 (11.6–12.3)	15.1 (14.8–15.5)	0.4 (0.3–0.5)	42.4 (41.5–43.4)	9.0 (8.6–9.3)	0.6 (0.5–0.7)	0.1 (0.0–0.2)

Middle aged adults from 35 to 60 years had second highest incidence rates with a total of 6.7 [6.7–6.8] ranging from 5.1 [4.9–5.4] in 2006 to 9.0 [8.6–9.3] in 2022. Children from 0 to 14 years (0.8 [0.8–0.9]), older adults from 60 to 79 (0.4 [0.4–0.4]) and elderly people over 80 years (0.1 [0.0-0.1]) showed only a very low incidence of OS-associated procedures. The total standardized incidence rate of OS-associated procedures in the maxilla was 4.8 per 100,000 person years [95% CI: 4.8–4.9] ranging from 3.3 [3.2–3.4] in 2005 to 6.2 [6.0–6.3] in 2022, an increase of + 88.2% within the observational period. In the mandible the total incidence rate was 6.3 [6.3–6.4] varying between 5.1 [5.0-5.3] in 2005 and 7.5 [7.3–7.6] in 2022, representing an increase of + 45.0% within the observational period. Looking at the 16 federal states individually the overall incidence of surgical procedures associated with OS within the observational period ranged from 5.3 [5.1–5.7] in Brandenburg to 22.3 [22.0-22.6] in Hesse (Supplementary Table 1). A single year minimum was seen in Brandenburg in 2005 with 2.7 [2.0-3.3]. A single year maximum was registered in Hesse with 28.0 [26.7–29.3] in 2021.

**Fig. 4:**
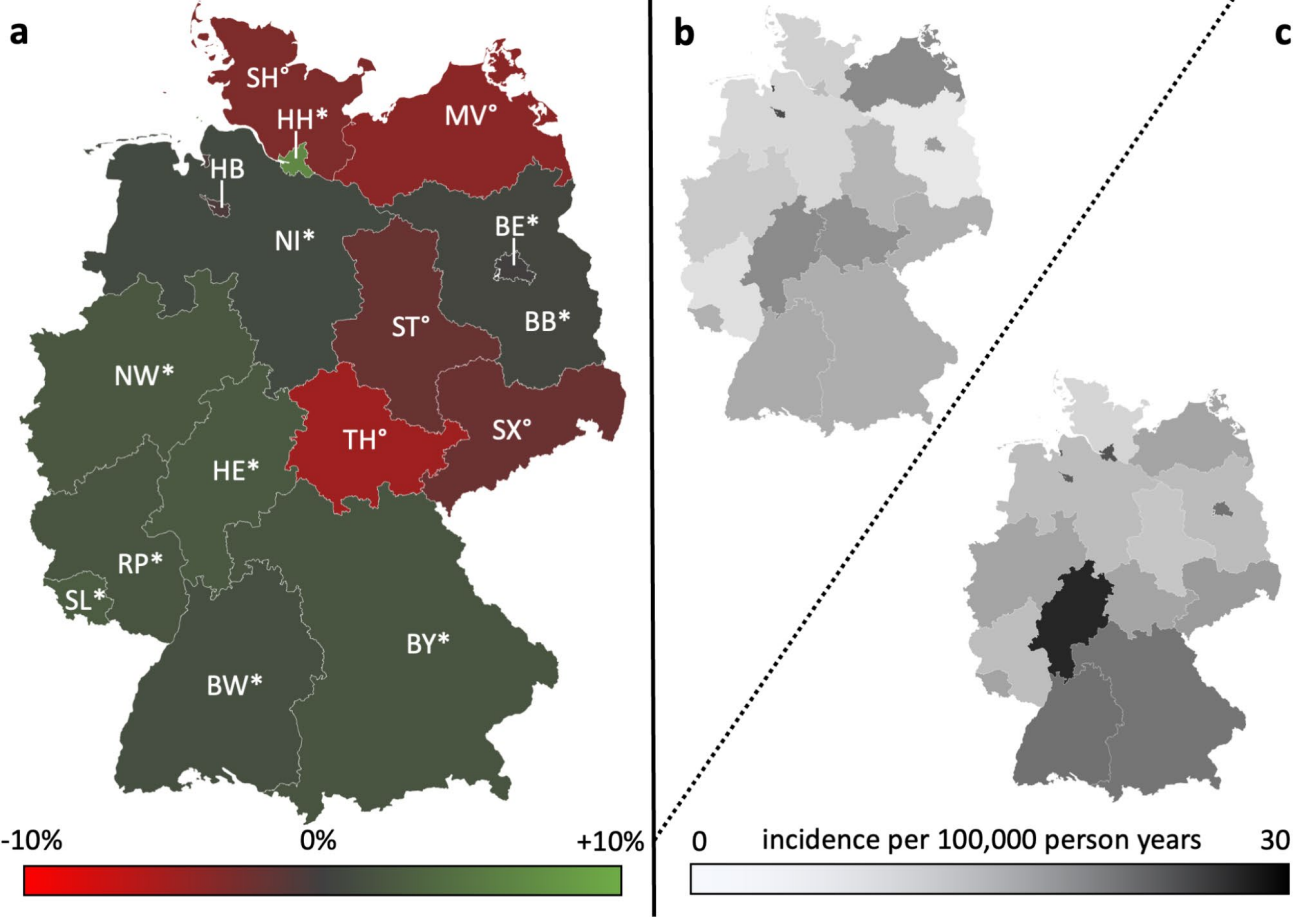
**A**: Change of the incidence of OS-associated procedures. Annual percentage change as result from the fully adjusted Poisson models stratified by federal state. Germany, 2005–2022. *Significant increase (p-value time trend Poisson-Regression model < 0.05); °Significant decrease (p-value time trend Poisson-Regression model < 0.05) SH: Schleswig Holstein; MV: Mecklenburg-Vorpommern; HH: Hamburg; HB: Bremen; NI: Lower saxony; BE: Berlin; BB: Brandenburg; ST: Saxony-Anhalt; NW: North Rhine-Westphalia; HE: Hesse; TH: Thuringia; SX: Saxony; RP: Rhineland-Palatinate; SL: Saarland; BW: Baden-Wurttemberg; BY: Bavaria. **B**: Baseline incidence of OS-associated procedures standardized for age and gender, Germany, 2005. **C**: Latest Incidence of OS-associated procedures standardized for age and gender, Germany, 2022.

The results of the time trend of incidence from the fully adjusted Poisson models are shown in Tables 3 and 4; Fig. 4. We observed a marked increase in the incidence of OS-associated procedures with a significant annual percentage increase of 2.5% in the observational period (relative risk per calendar year 1.025; 95% CI: 1.024–1.025, p-value < 0.001). The increase was slightly weaker in females (2.1% per year, RR: 1.021; 1.020–1.021; p-value < 0.001) than in males (3.0% per year, RR:1.030; 1.030–1.031; *p* < 0.001), although the baseline incidence was higher in females. Significant increases were observed in age groups 15–34 years (2.6% per year, RR: 1.026; 1.025–1.026; *p* < 0.001), 35–59 years (2.4% per year, RR: 1.024; 1.023–1.025; *p* < 0.001) and 60–79 years (0.6% per year, RR: 1.006; 1.003–1.009; *p* < 0.001). In contrast a significant decrease was seen in children from 0 to 14 years (-2.9% per year, RR: 0.971; 0.968–0.974; *p* = 0.001). A significant increase of OS-associated procedures was found in 10 of 16 federal states ranging from 1.3% per year in Berlin (RR: 1.013; 1.010–1.017; *p* < 0.001) to 7.1% per year in Hamburg (RR: 1.071; 1.064–1.077 ; *p* < 0.001).

**Table 3:** Results of the Poisson models for total population and stratified by gender and age group (Germany, 2005–2022). Relative risk of OS (95% confidence interval). * p-value < 0.05; ^a^ baseline: gender: female, age:35–59 years; ^b^ baseline: age:35–59 years.

Population	Total	Age group				
		0–14	15–34	35–59	60–79	80+
All						
Calendar year	1.025 (1.024–1.025)*	0.971 (0.968–0.974)*	1.026 (1.025–1.026)*	1.024 (1.023–1.025)*	1.006 (1.003–1.009)*	0.981 (0.962–0.999)*
Gender^a^	0.666 (0.664–0.668)*	0.752 (0.729–0.776)*	0.702 (0.699–0.704)*	0.536 (0.532–0.540)*	1.035 (1.000–1.072)*	1.653 (1.356–2.014)*
Age group		0.123 (0.121–0.125)*	5.521 (5.501–5.542)*	1.000^a^	0.060 (0.059–0.062)*	0.006 (0.006–0.007)*
Males						
Calendar year	1.030 (1.030–1.031)*	0.969 (0.964–0.973)*	1.031 (1.031–1.032)*	1.031 (1.030–1.032)*	0.988 (0.983–0.993)*	1.018 (0.989–1.048)
Age group		0.151 (0.148–0.155)*	6.509* (6.469–6.549)*	1.000^b^	0.089 (0.087–0.091)*	0.013 (0.011–0.015)*
Females						
Calendar year	1.021 (1.020–1.021)*	0.973 (0.969–0.977)*	1.021 (1.021–1.022)*	1.020 (1.020–1.021)*	1.023 (1.018–1.028)*	0.950 (0.926–0.975)*
Age group		0.108 (0.106–0.110)*	4.976 (4.953–4.999)*	1.000^b^	0.046 (0.045–0.047)*	0.004 (0.004–0.005)*
Procedures in the maxilla						
Calendar year	1.034 (1.034–1.035)*	0.992 (0.988–0.995)*	1.034 (1.034–1.035)*	1.039 (1.038–1.040)*	1.032 (1.026–1.038)*	0.968 (0.933–1.004)*
Gender^a^	0.756 (0.752–0.759)*	0.778 (0.749–0.807)*	0.795 (0.790–0.799)*	0.608 (0.601–0.614)*	0.883 (0.832–0.938)*	4.088 (2.719–6.148)*
Age group		0.215 (0.211–0.219)*	6.352* (6.314–6.390)*	1.000^a^	0.053 (0.051–0.059)*	0.005 (0.004–0.006)*
Procedures in the mandible						
Calendar year	1.017 (1.017–1.017)*	0.935 (0.930–0.939)*	1.019 (1.018–1.019)*	1.014 (1.014–1.015)*	0.994 (0.990–0.998)*	0.942 (0.920–0.965)*
Gender^a^	0.604 (0.601–0.606)*	0.715 (0.678–0.753)*	0.634 (0.631–0.637)*	0.497 (0.493–0.501)*	1.098 (1.052–1.145)*	1.557 (1.214–1.998)*
Age group		0.069 (0.067–0.070)*	4.995 (4.971–5.019)*	1.000^a^	0.066 (0.064–0.067)*	0.007 (0.006–0.007)*

A significant decrease of OS-associated procedures was found in 5 of 16 federal states ranging from − 1.0% per year in Saxony-Anhalt (RR: 0.990; 0.984–0.996; *p* < 0.001) to -4.3% per year in Thuringia (RR: 0.957; 0.951–0.963 ; *p* < 0.001). There was no significant change in Bremen. Correlation analysis revealed strong, statistically highly significant correlations between the Incidence of OS-associated procedures I_OS_ and regional HDI (*r* = 0,830; *p* < 0.01) as well as regional gross domestic product (GDP) per capita (*r* = 0,761; *p* < 0.01; Table 5).

**Table 4:** Results of the Poisson models stratified by federal state (Germany, 2005–2022). Relative risk of OS (95% confidence interval). * p-value < 0.05; ^a^ baseline: gender: female, age:35–59 years.

	Federal state							
	Baden-Württemberg	Bavaria	Berlin	Brandenburg	Bremen	Hamburg	Hesse	Mecklenburg-Vorpommern
Risk factor								
Calendar year	1.026 (1.024–1.029)*	1.030 (1.028–1.033)*	1.013 (1.010–1.017)*	1.019 (1.011–1.028)*	1.006 (0.999–1.013)	1.071 (1.064–1.077)*	1.034 (1.031–1.036)*	0.969 (0.962–0.975)*
Gender^**a**^	0.726 (0.708–0.745)*	0.708 (0.692–0.726)*	0.736 (0.706–0.766)*	0.573 (0.523–0.627)*	0.672 (0.625–0.722)*	0.714 (0.671–0.759)*	0.696 (0.679–0.714)*	0.670 (0.626–0.717)*

	Federal state							
	Lower saxony	North Rhine-Westphalia	Rhineland-Palatinate	Saarland	Saxony	Saxony-Anhalt	Schleswig-Holstein	Thuringia
Risk factor								
Calendar year	1.022 (1.018–1.026)*	1.032 (1.030–1.034)*	1.030 (1.024–1.036)*	1.036 (1.026–1.046)*	0.989 (0.984–0.994)*	0.990 (0.984–0.996)*	0.977 (0.970–0.984)*	0.957 (0.951–0.963)*
Gender^**a**^	0.725 (0.695–0.756)*	0.699 (0.683–0.715)*	0.746 (0.703–0.792)*	0.660 (0.596–0.732)*	0.812 (0.774–0.853)*	0.595 (0.555–0.638)*	0.700 (0.649–0.756)*	0.725 (0.682–0.771)*

**Fig. 5:**
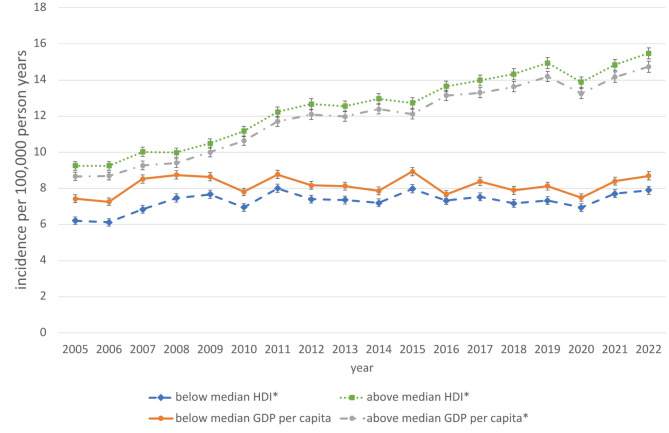
Standardized age-specific, regional incidence of OS-associated procedures stratified by regional HDI and regional GDP per capita, Germany, 2005–2022. *Significant increase (p value time trend Poisson-Regression model < 0.05).

Moreover, a strong significant correlation was found with physician density per capita (*r* = 0,640; *p* = 0.023) and dentist density per capita (*r* = 0,590; *p* = 0.036). Regarding the annual change in the regional incidence of OS-associated procedures 2005–2022 ΔI_OS_ correlation analysis revealed strong, statistically significant correlations with the regional HDI (*r* = 0,659; *p* = 0.017), the regional GDP per capita (*r* = 0,730; *p* = 0.012) and the regional median household income (*r* = 0,662; *p* = 0.023). There was no statistically significant correlation to any other investigated demographic parameter. Stratified by HDI there is a marked significant increase of 3.0% per year (RR: 1.030; 1.028–1.031; *p* < 0.001) of OS-associated procedures in federal states with HDI above the median HDI compared to 0.8% annual increase in federal states below the median HDI (RR: 1.008; 1.006–1.010 ; *p* < 0.001; Fig. 5). Stratified by GDP per capita there is significant increase of 3.0% per year (RR: 1.030; 1.029–1.031; *p* < 0.001) of OS-associated procedures in federal states with GDP per capita above the median compared to a non-significant annual increase of 0,2% in federal states below the median GDP per capita (RR: 1.002; 0.999–1.004 ; *p* = 0,14).

**Table 5:** Pearson correlation r_p_ between regional demographic parameters 2022 and the regional incidence of OS 2022 I_OS_ as well as the annual change in the regional incidence of OS 2005–2022 ΔI_OS_.

	Demographic parameters 2022	Median [Range]	I_OS_		ΔI_OS_	
			*r*_*p*_	*p*-value	*r*_*p*_	*p*-value
Population	Population density (inhabitants per km^2^)	215 [70–4214]	0,451	0,102	0,300	0,465
	Human development index (HDI)	0.941 [0.921–0.975]	**0**,**790**	**< 0**,01*	**0**,**659**	**0**,017*
Economy	Gross domestic product per capita (1000 €)	43.2 [35.7–79.2]	**0**,**761**	**< 0**,01*	**0**,**730**	**0**,012*
	Median household income (1000 €)	21.7 [19.5–25.3]	0,504	0,084	**0**,**662**	**0**,023*
Healthcare	Life expectancy (years)	82.97 [82.06–84.11]	0,366	0,184	0,640	0,487
	Physician density (per 100,000)	217 [199–306]	**0**,**640**	**0**,023*	0,370	0,355
	Dentist density (per 100,000)	83 [70–114]	**0**,**590**	**0**,036*	0,294	0,404
	OMF-specialist density (per 100,000)	1.6 [0.6–4.0]	0,473	0,096	0,235	0,430
	Density of hospital beds (per 100,000)	600 [478–715]	0,032	0,905	-0.177	0,512

## Discussion

Orthognathic surgery has evolved considerably in recent decades, with advances reducing morbidity and expanding indications, making patients receiving orthognathic surgery an important group of hospital inpatients in oral and maxillofacial surgery departments^1,3–5^. Recent trends include greater precision through computer-aided planning and simulation as well as patient-specific fixation, making orthognathic surgery a highly predictable and increasingly sought-after treatment option for dentofacial anomalies and obstructive sleep disorders^12^. However, reliable population-based data about long-term trends, age and gender distributions in the incidence of OS-associated procedures are limited. Regional data may be inconclusive due to the heterogenic concentration on a smaller number of not equally distributed surgical centers. Most available data was collected from single center case series and therefore may not be representative. To our knowledge, this is the first population-based study to investigate the incidence of OS-associated procedures using Germany’s DRG inpatient billing data base, which includes data on the entire population of Germany.

Within the observational period from 2005 to 2022, we found a mean incidence of OS-associated procedures 11.1 / 100,000 person years in Germany. As in our case with a gender ratio of 1:1.4 a number of previous studies have shown a moderate preponderance of orthognathic surgery in women (13.0 /100,00 person years) compared to men (9.2 / 100,000 person years)^18,26–28^. The observed trend is believed to reflect female patients’ desire for aesthetic improvement, as orthognathic surgery not only improves functional impairment but also addresses many aspects of facial aesthetics. Moreover, in comparison to the male-to-female ratio of 1:1.45 observed during the initial half of the study period, the proportion of male patients who underwent orthognathic surgery increased during the subsequent half (male-to-female ratio 1:1.38). This finding reflects a recent marked increase in aesthetic and functional awareness and a narrowing difference between males and females^18,27,28^.

Overall, we were able to detect a significant increase in the incidence of OS-associated procedures within the observational period in Germany (+ 2.5% per year) affecting both genders (♀+2.1 per year; ♂+3.0% per year) in similar fashion. These results were in accordance with other data from different countries describing an increase in orthognathic surgery procedures. Moles et al. report a gradual increase in the number of procedures each year from 855 in 1997 to 1237 in 2005 in the hospitals of the National Health System in England^29^. Hamada et al. show an increasing incidence of orthognathic surgeries at the hospital of the Tokyo Dental College in Japan rising from 7 cases per year in 1990 to more than 150 cases per year in 2016^18^. Similar data was published by Lee et al. describing an increase in OS between 2004 and 2015 at the National University Dental Hospital in Seoul, South Korea^19^.

This phenomenon can, to some extent, be attributed to the increasing societal acceptance and legitimization of aesthetic procedures^18,29^. In recent years there is an increased attention for facial aesthetics and dental aesthetics through social media and the increase in work-related online video communication^30,31^. Advancements in orthodontic and surgical techniques, coupled with the reduced morbidity associated with orthognathic interventions, may have influenced the growing inclination of clinicians to recommend such treatments and patients to pursue them^18,29^. Enhanced accessibility to these procedures in certain regions may also play a contributing role. The field of orthognathic surgery has witnessed substantial progress over the past century, with particularly rapid advancements in the last two decades. Innovations in imaging technologies, computer-assisted surgical planning, patient-specific fixation methods, surgery first concepts and a deeper understanding of the pathophysiology of airway obstruction have empowered surgeons to address a broader spectrum of diagnoses and to effectively manage even the most complex anatomical cases^12^. Nevertheless, some studies have documented a decreasing incidence of OS in specific centers or regional assessments especially in the USA, where the reduction in monetary reimbursement from insurance providers might be one major reason for the reduction in the number of surgical procedures^19–21^. The increase in the incidence of OS-associated procedures within the observational period is not equally addressable to both the maxilla and the mandible. While the increase in maxillary procedures is rising with 3.4% per year, mandibular procedures show a weaker increase with 1.7% per year showing an ongoing trend to bimaxillary surgery as indicated in recent studies. While single-jaw surgeries were once common, there has been a shift towards maxillary advancement and combined maxillary-mandibular procedures particularly for Class III malocclusions as well as aesthetically driven bimaxillary surgery in Class II malocclusions^32,33^. Bimaxillary surgeries now constitute the largest group of orthognathic procedures in most centers^34^. Despite the increased complexity, when indicated, bimaxillary surgery offers more surgical options and potentially better results than single-jaw procedures^35^.

Looking at the different age groups we found the strongest increase in adolescents and young adults form 15–34 years (+ 2,6% per year) followed by adults from 35 to 59 years (+ 2.4% per year) and older adults from 60 to 79 years (+ 0.6% per year). Pediatric patients aged 0–14 years (-2.9% per year) show a significant decrease in the incidence of OS-associated procedures. For people over 80 years, orthognathic surgery was and still is a marginal phenomenon and is reserved for a few rare indications. The average age at the time of orthognathic surgery was 27.6 years, rising from 27.2 years in 2005 to 29.0 years in 2022. The proportion of patients who had undergone surgery by the age of 35 remains between 75.9% and 79.8% throughout the observational period, indicating that the age group of adolescents and young adults from 15 to 34 years remains the most prevalent group. However, the number of patients undergoing orthognathic surgery between the ages of 35 and 79 has markedly increased in recent years. The most important factors influencing this trend are the increased attention for facial aesthetics, dental aesthetics and combined surgical and orthodontic treatment caused by the various new and social media and progress in the field of facial rejuvenation^30,31,36^. Moreover, OS is becoming increasingly important in the treatment of sleep-related breathing disorders or for pre-prosthetic occlusion improvement in implantology. It can be assumed that orthognathic surgery for this type of patients will continue to increase^12,18^. The reduction in the number of procedures performed on children may be attributed to advancements in orthodontic treatment techniques, including e.g. the utilization of orthodontic mini-implants and skeletal-anchored orthodontics. These developments may have diminished the necessity for surgical interventions, such as the common surgically assisted rapid maxillary expansion^37^.

It is evident that there are discernible disparities in the regional incidence of orthognathic surgery and its evolution over time. The incidence of orthognathic surgery is markedly higher and, most notably, has increased at a more pronounced rate over time in economically robust federal states with higher individual income. While in half of the federal states with a higher gross domestic product per capita there was a clear increase in the frequency of orthognathic interventions of 3% per year, in the other half there was no significant change over time.

In a national registry-based study in Sweden, Stålhand et al. identified significant regional variations in the prevalence of orthognathic surgery. These variations were associated with demographic differences. The researchers hypothesized that there are differences in the regional traditions used to assess indications for treatment and in the accessibility of surgical facilities^38^. In Scotland, patients living farther from hospitals were less likely to present for minor malocclusions, suggesting distance may influence access to care^39^. Socioeconomic factors also play a role, as a study in England revealed that most patients receiving orthognathic treatment lived in relatively affluent areas, indicating potential inequalities in access^26^. However, a US study found no evidence of concentration of orthognathic procedures in teaching hospitals over time, suggesting that regionalization to specific hospital types may not be a significant factor^40^. These findings highlight the complex interplay of geographic, socioeconomic, and healthcare system factors in orthognathic surgery distribution.

This study is subject to certain strengths and limitations, which are mainly related to the dataset that was available for analysis. Firstly, it should be noted that the specific reimbursed OPS codes from the national DRG system database only represent OS treated on an inpatient basis in the analyzed years. OS done on an outpatient basis without hospital admission or using other reimbursement schemes than the DRG-system could not be included in the dataset. Since statutory health insurance in Germany provides a fully accessible and affordable healthcare system for every citizen and OS is covered in the vast majority of indications, we assume that our study can cover the vast majority of the German population and provide a comprehensive overview. Selection bias in patient inclusion due to socioeconomic status is limited by the population-based approach. The completeness of our description is only limited by the dataset itself, which provides information at only an aggregated level and in limited detail without information about the specific kind of dentofacial impairments or severity of the disorder. The sample also includes orthognathic interventions related to cleft repair, correction of craniosynostosis, distraction osteogenesis procedures related to hemifacial microsomia or Pierre Robin syndrome, or secondary interventions related to trauma but does not allow us to quantify these cases in the total sample, which would be highly interesting. Consequently, conducting a more advanced analysis encompassing more detailed medical information was not feasible. However, further exploration into the reasons for the increasing numbers of OS would be a worthwhile endeavor. Nonetheless, the DRG inpatient billing database offers an exceptional instrument for German researchers to undertake clinical epidemiology investigations on a larger scale regarding procedures and diseases^41^. The utilization of claims data has been demonstrated to circumvent the recall and misclassification biases that may emerge in studies reliant upon self-reported data.

This study is, to our knowledge, the first to examine the temporal course of the incidence and epidemiologic distribution of OS at a national level in Germany. The analysis of the influence of population structure on the implementation of OS can be facilitated by this approach. A comprehensive description of these effects will facilitate the identification of groups that stand to benefit from future consultation regarding OS. To this end, the investigation of the direct influence of social and economic background should be expanded.

## Conclusion

Orthognathic surgery has seen significant advancements in precision and accessibility, driven by technological innovations and societal shifts towards greater acceptance of aesthetic procedures. This population-based study, the first of its kind in Germany, reveals a steady increase in the incidence of OS-associated procedures between 2005 and 2022, with an annual increase of 2.5%, especially among adolescents, young adults, and middle-aged patients. This expansion is attributed to advancements in surgical precision, rising awareness regarding orofacial function and aesthetics and a wider range of clinical indications for example in the treatment of sleep-related breathing disorders. The incidence remains slightly higher among women, while acceptance among men has increased in recent years. The declining incidence in pediatric patients may underline the effectiveness of modern orthodontic treatments but requires further research to elucidate this decline.

It is highly noteworthy that the above-mentioned effects are limited to economically affluent regions, reflecting the impact of socioeconomic and regional factors on healthcare accessibility. Disparities persist due to socioeconomic and regional differences in access to care, emphasizing the need for targeted strategies to address underserved populations. Future studies should analyze the influence of socioeconomic factors and integrate diagnostic data to improve accessibility to OS. While limited by data scope, this research highlights the importance of comprehensive datasets for understanding trends and informing future healthcare planning.

## Electronic supplementary material

Below is the link to the electronic supplementary material.

Supplementary Material 1

## Data Availability

The datasets generated during and analyzed during the current study are available from the corresponding author on reasonable request.
