# Supplementary material for: Epidemiologic trends and increasing inequalities in orthognathic surgery in Germany: a nationwide analysis 2005–2022
Source: Sci Rep. 2025 Feb 20;15:6223. doi: 10.1038/s41598-025-90889-1 (PMC11842797; doi:10.1038/s41598-025-90889-1)
Supplement: Supplementary file 1 — Supplementary Material 1 [file 41598_2025_90889_MOESM1_ESM.docx]

**Supplementary Information File for:**

Epidemiologic trends and increasing inequalities in orthognathic surgery in Germany: A nationwide analysis 2005 - 2022

Axel Meisgeier^1*^, Simon Pienkohs^1^, Constantin Salomia^1^, Laura Moosdorf^2^, Andreas Neff^1^

1. Department of Oral and Craniomaxillofacial Surgery, UKGM GmbH, University Hospital Marburg and Faculty of Medicine, Philipps University, Marburg, Germany
2. Center for Orthopaedics and Trauma Surgery, UKGM GmbH, University Hospital Marburg and Faculty of Medicine, Philipps University, Marburg, Germany

Corresponding author*

Dr. Dr. Axel Meisgeier

Department of Oral and Craniomaxillofacial Surgery

UKGM GmbH, University Hospital Marburg

Faculty of Medicine, Philipps University, Marburg

35043 Marburg, Germany

e-mail: axel.meisgeier@med.uni-marburg.de

phone: +49 (0)6421 58-63237
fax: +49 (0)6421 58-68990

This document includes Supplementary table 1.

**Supplementary Table 1:** Regional incidence of OS-associated procedures in all 16 federal states of Germany 2005 - 2022. Incidence rates (95 % confidence interval) per 100,000 person years standardized to the German population based on the Census 2011.

|  | Germany | Baden- Wuerttemberg | Bavaria | Berlin | Brandenburg | Bremen | Hamburg | Hesse | Mecklenburg- Vorpommern | Lower Saxony | North Rhine- Westphalia | Rhineland- Palatinate | Saarland | Saxony | Saxony- Anhalt | Schleswig- Holstein | Thuringia |
| --- | --- | --- | --- | --- | --- | --- | --- | --- | --- | --- | --- | --- | --- | --- | --- | --- | --- |
| **All Years** | **11.1 (11.1 – 11.2)** | 11.9 (11.7 - 12.0) | 12.0 (11.8 - 12.1) | 13.8 (13.5 - 14.0) | 5.3 (5.1 - 5.6) | 21.1 (20.3 - 21.9) | 13.2 (12.8 - 13.6) | 22.3 (22.0 - 22.6) | 12.0 (11.6 - 12.4) | 6.3 (6.1 - 6.4) | 9.6 (9.5 - 9.7) | 6.0 (5.9 - 6.2) | 9.2 (8.8 - 9.7) | 9.7 (9.5 - 10.0) | 9.2 (8.9 - 9.5) | 5.7 (5.5 - 5.9) | 11.6 (11.3 - 12.0) |
| **2005-2013** | **9.8 (9.8 - 9.9)** | 10.6 (10.4 - 10.8) | 10.3 (10.1 - 10.5) | 13.3 (12.9 - 13.7) | 4.4 (4.1 - 4.7) | 20.6 (19.4 - 21.7) | 9.4 (8.9 - 9.9) | 18.6 (18.2 - 19.0) | 13.5 (12.9 - 14.1) | 5.9 (5.7 - 6.0) | 8.1 (8.0 - 8.2) | 4.8 (4.6 - 5.1) | 7.5 (7.0 - 8.1) | 9.9 (9.6 - 10.3) | 9.0 (8.6 - 9.4) | 6.4 (6.0 - 6.7) | 13.7 (13.2 - 14.2) |
| **2014-2022** | **12.4 (12.4 - 12.5)** | 13.1 (12.9 - 13.4) | 13.7 (13.4 - 13.9) | 14.2 (13.8 - 14.6) | 6.3 (6.0 - 6.6) | 21.6 (20.4 - 22.7) | 17.0 (16.4 - 17.7) | 26.0 (25.6 - 26.5) | 10.5 (10.0 - 11.0) | 6.7 (6.5 - 6.9) | 11.2 (11.0 - 11.3) | 7.3 (7.0 - 7.5) | 10.9 (10.2 - 11.6) | 9.5 (9.2 - 9.8) | 9.4 (9.0 - 9.8) | 5.1 (4.8 - 5.4) | 9.6 (9.1 - 10.0) |
| **RR** | **1.27 (1.25 - 1.28)*** | 1.24 (1.20 - 1.27)* | 1.32 (1.29 - 1.36)* | 1.07 (1.02 - 1.11)* | 1.43 (1.32 - 1.55)* | 1.05 (0.97 - 1.13) | 1.81 (1.70 - 1.93)* | 1.40 (1.36 - 1.43)* | 0.78 (0.73 - 0.83)* | 1.14 (1.09 - 1.19)* | 1.38 (1.35 - 1.41)* | 1.50 (1.41 - 1.59)* | 1.45 (1.31 - 1.59)* | 0.96 (0.91 - 1.00) | 1.04 (0.98 - 1.11) | 0.80 (0.74 - 0.86)* | 0.70 (0.66 - 0.74)* |
| 2005 | **8.4 (8.2 - 8.6)** | 9.9 (9.3 - 10.5) | 9.6 (9.0 - 10.1) | 11.6 (10.5 - 12.8) | 2.7 (2.0 - 3.3) | 21.3 (17.8 - 24.8) | 7.8 (6.5 - 9.2) | 13.5 (12.6 - 14.5) | 13.7 (11.9 - 15.4) | 4.7 (4.3 - 5.2) | 6.2 (5.9 - 6.6) | 3.6 (3.0 - 4.2) | 9.1 (7.3 - 11.0) | 9.4 (8.5 - 10.3) | 8.6 (7.4 - 9.7) | 5.4 (4.6 - 6.3) | 12.9 (11.4 - 14.3) |
| 2006 | **9.1 (8.9 - 9.3)** | 9.3 (8.7 - 9.9) | 8.4 (7.9 - 8.9) | 11.4 (10.3 - 12.5) | 3.6 (2.9 - 4.3) | 18.4 (15.1 - 21.6) | 7.1 (5.8 - 8.3) | 16.9 (15.8 - 17.9) | 13.4 (11.6 - 15.1) | 4.8 (4.3 - 5.3) | 6.6 (6.2 - 7.0) | 3.5 (2.9 - 4.0) | 6.8 (5.3 - 8.4) | 9.4 (8.4 - 10.3) | 8.3 (7.1 - 9.4) | 5.7 (4.8 - 6.6) | 11.8 (10.4 - 13.2) |
| 2007 | **9.3 (9.1 - 9.5)** | 10.5 (9.9 - 11.1) | 8.9 (8.4 - 9.4) | 10.8 (9.7 - 11.9) | 3.8 (3.1 - 4.6) | 21.2 (17.7 - 24.7) | 6.3 (5.1 - 7.4) | 18.0 (16.9 - 19.1) | 12.4 (10.8 - 14.1) | 5.3 (4.8 - 5.8) | 7.0 (6.6 - 7.4) | 4.7 (4.0 - 5.4) | 8.3 (6.5 - 10.0) | 12.3 (11.2 - 13.3) | 9.6 (8.4 - 10.8) | 7.1 (6.1 - 8.1) | 12.5 (11.0 - 13.9) |
| 2008 | **9.7 (9.5 - 9.9)** | 10.0 (9.4 - 10.6) | 9.3 (8.8 - 9.8) | 12.6 (11.4 - 13.8) | 3.1 (2.4 - 3.8) | 17.8 (14.6 - 21.0) | 6.0 (4.9 - 7.2) | 16.6 (15.5 - 17.6) | 15.4 (13.5 - 17.3) | 5.9 (5.4 - 6.4) | 7.5 (7.1 - 7.9) | 5.5 (4.8 - 6.2) | 6.2 (4.7 - 7.7) | 10.8 (9.8 - 11.8) | 9.1 (7.9 - 10.3) | 6.6 (5.7 - 7.6) | 16.7 (15.0 - 18.3) |
| 2009 | **10.0 (9.8 - 10.2)** | 9.5 (8.9 - 10.1) | 9.9 (9.4 - 10.5) | 14.4 (13.1 - 15.7) | 5.2 (4.3 - 6.1) | 20.7 (17.2 - 24.2) | 7.9 (6.6 - 9.2) | 18.0 (16.9 - 19.0) | 12.4 (10.7 - 14.1) | 6.6 (6.0 - 7.1) | 8.0 (7.6 - 8.4) | 5.6 (4.8 - 6.3) | 5.8 (4.3 - 7.3) | 10.3 (9.4 - 11.3) | 8.4 (7.3 - 9.6) | 7.1 (6.2 - 8.1) | 16.3 (14.6 - 18.0) |
| 2010 | **11.1 (10.8 - 11.3)** | 10.6 (10.0 - 11.3) | 11.3 (10.7 - 11.9) | 13.6 (12.4 - 14.8) | 4.6 (3.7 - 5.4) | 18.5 (15.2 - 21.8) | 10.1 (8.6 - 11.5) | 19.3 (18.2 - 20.4) | 12.5 (10.8 - 14.2) | 6.2 (5.7 - 6.8) | 8.2 (7.7 - 8.6) | 4.2 (3.5 - 4.8) | 7.7 (6.0 - 9.4) | 9.8 (8.9 - 10.8) | 8.7 (7.5 - 9.9) | 5.6 (4.7 - 6.5) | 13.7 (12.1 - 15.2) |
| 2011 | **11.3 (11.0 - 11.5)** | 11.5 (10.9 - 12.2) | 11.7 (11.1 - 12.3) | 15.2 (13.9 - 16.5) | 7.8 (6.7 - 9.0) | 22.1 (18.5 - 25.7) | 12.4 (10.7 - 14.0) | 21.5 (20.3 - 22.6) | 13.2 (11.4 - 15.0) | 6.9 (6.3 - 7.5) | 9.4 (8.9 - 9.8) | 5.2 (4.5 - 5.9) | 7.9 (6.2 - 9.7) | 9.7 (8.8 - 10.7) | 8.9 (7.7 - 10.2) | 6.8 (5.8 - 7.8) | 14.6 (13.0 - 16.2) |
| 2012 | **11.2 (11.0 - 11.4)** | 12.0 (11.3 - 12.6) | 12.2 (11.6 - 12.8) | 14.9 (13.6 - 16.2) | 5.0 (4.1 - 5.9) | 22.6 (18.9 - 26.2) | 13.1 (11.4 - 14.8) | 23.1 (21.9 - 24.3) | 15.3 (13.4 - 17.2) | 6.2 (5.7 - 6.8) | 9.7 (9.3 - 10.2) | 5.3 (4.5 - 6.0) | 8.4 (6.6 - 10.1) | 8.9 (8.0 - 9.8) | 10.0 (8.7 - 11.3) | 6.3 (5.3 - 7.2) | 11.5 (10.0 - 12.9) |
| 2013 | **11.5 (11.2 - 11.7)** | 12.3 (11.7 - 13.0) | 11.6 (11.0 - 12.2) | 15.4 (14.1 - 16.7) | 3.9 (3.1 - 4.7) | 22.7 (19.0 - 26.3) | 13.9 (12.1 - 15.6) | 20.7 (19.5 - 21.8) | 13.3 (11.6 - 15.1) | 6.1 (5.6 - 6.7) | 10.2 (9.8 - 10.7) | 6.1 (5.3 - 6.8) | 7.6 (5.9 - 9.3) | 8.8 (7.9 - 9.7) | 9.2 (8.0 - 10.5) | 6.5 (5.6 - 7.5) | 13.4 (11.9 - 15.0) |
| 2014 | **11.5 (11.3 - 11.7)** | 11.6 (10.9 - 12.2) | 12.3 (11.7 - 12.9) | 15.3 (14.0 - 16.6) | 6.0 (5.0 - 6.9) | 24.9 (21.1 - 28.7) | 14.0 (12.3 - 15.8) | 22.8 (21.6 - 24.0) | 10.4 (8.8 - 12.0) | 5.8 (5.3 - 6.3) | 10.9 (10.5 - 11.4) | 7.0 (6.2 - 7.8) | 8.2 (6.4 - 10.0) | 7.7 (6.9 - 8.6) | 9.2 (8.0 - 10.5) | 5.3 (4.4 - 6.1) | 12.1 (10.7 - 13.6) |
| 2015 | **10.2 (10.0 – 10.4)** | 10.4 (9.8 - 11.0) | 11.9 (11.3 - 12.5) | 13.8 (12.6 - 15.0) | 7.7 (6.6 - 8.8) | 18.4 (15.1 - 21.6) | 15.4 (13.5 - 17.2) | 25.1 (23.9 - 26.4) | 11.5 (9.8 - 13.1) | 5.6 (5.1 - 6.1) | 10.7 (10.2 - 11.2) | 8.7 (7.8 - 9.6) | 10.3 (8.3 - 12.3) | 8.1 (7.2 - 9.0) | 12.0 (10.6 - 13.5) | 5.5 (4.6 - 6.3) | 11.5 (10.1 - 12.9) |
| 2016 | **10.5 (10.3 – 10.7)** | 11.9 (11.2 - 12.5) | 13.2 (12.5 - 13.8) | 13.8 (12.6 - 15.0) | 6.0 (5.0 - 7.0) | 19.6 (16.3 - 22.9) | 15.9 (14.0 - 17.7) | 26.6 (25.3 - 27.9) | 8.3 (6.9 - 9.8) | 6.9 (6.3 - 7.4) | 11.3 (10.8 - 11.8) | 7.4 (6.6 - 8.2) | 8.9 (7.1 - 10.8) | 8.2 (7.3 - 9.0) | 8.0 (6.8 - 9.1) | 6.4 (5.5 - 7.3) | 9.6 (8.3 - 10.9) |
| 2017 | **10.7 (10.5 – 10.9)** | 12.6 (12.0 - 13.3) | 13.2 (12.6 - 13.8) | 13.2 (12.0 - 14.3) | 6.5 (5.5 - 7.5) | 20.6 (17.2 - 24.0) | 17.2 (15.3 - 19.1) | 27.7 (26.4 - 29.0) | 10.9 (9.3 - 12.5) | 6.2 (5.7 - 6.8) | 11.2 (10.7 - 11.7) | 7.0 (6.2 - 7.9) | 13.4 (11.1 - 15.6) | 9.2 (8.3 - 10.2) | 10.4 (9.0 - 11.7) | 6.9 (5.9 - 7.9) | 7.4 (6.2 - 8.5) |
| 2018 | **11.1 (10.8 – 11.3)** | 13.3 (12.7 - 14.0) | 13.2 (12.6 - 13.8) | 12.3 (11.1 - 13.4) | 5.1 (4.2 - 6.0) | 23.8 (20.2 - 27.5) | 17.7 (15.8 - 19.6) | 27.7 (26.4 - 29.0) | 9.4 (7.9 - 10.9) | 6.7 (6.1 - 7.2) | 11.8 (11.3 - 12.3) | 6.6 (5.8 - 7.3) | 11.4 (9.3 - 13.5) | 9.9 (8.9 - 10.9) | 10.9 (9.5 - 12.3) | 4.6 (3.8 - 5.4) | 8.9 (7.6 - 10.1) |
| 2019 | **11.6 (11.4 – 11.8)** | 13.1 (12.4 - 13.8) | 15.3 (14.7 - 16.0) | 14.2 (13.0 - 15.5) | 5.8 (4.9 - 6.8) | 24.2 (20.5 - 27.9) | 18.2 (16.3 - 20.2) | 26.0 (24.7 - 27.2) | 12.3 (10.6 - 14.1) | 6.7 (6.1 - 7.2) | 12.4 (11.9 - 13.0) | 7.5 (6.6 - 8.3) | 9.7 (7.7 - 11.6) | 10.1 (9.1 - 11.1) | 9.3 (8.1 - 10.6) | 3.9 (3.2 - 4.6) | 9.1 (7.8 - 10.4) |
| 2020 | **11.0 (10.8 – 11.2)** | 14.7 (14.0 - 15.4) | 13.5 (12.9 - 14.1) | 13.7 (12.5 - 14.9) | 4.3 (3.5 - 5.1) | 18.6 (15.4 - 21.8) | 16.2 (14.4 - 18.1) | 24.8 (23.6 - 26.1) | 10.3 (8.8 - 11.9) | 6.8 (6.3 - 7.4) | 10.4 (9.9 - 10.9) | 5.7 (4.9 - 6.4) | 14.7 (12.3 - 17.1) | 9.5 (8.5 - 10.4) | 8.0 (6.8 - 9.1) | 4.1 (3.3 - 4.8) | 9.9 (8.6 - 11.2) |
| 2021 | **10.5 (10.3 – 10.8)** | 14.0 (13.3 - 14.7) | 14.1 (13.4 - 14.7) | 14.8 (13.6 - 16.1) | 7.4 (6.3 - 8.4) | 24.8 (21.0 - 28.5) | 18.5 (16.6 - 20.5) | 28.0 (26.7 - 29.3) | 10.7 (9.1 - 12.3) | 7.6 (7.0 - 8.2) | 11.4 (10.9 - 11.9) | 7.7 (6.9 - 8.6) | 11.0 (8.9 - 13.0) | 11.1 (10.0 - 12.1) | 9.9 (8.5 - 11.2) | 4.2 (3.5 - 5.0) | 7.1 (6.0 - 8.3) |
| 2022 | **11.1 (10.8 – 11.3)** | 16.7 (15.9 - 17.4) | 16.3 (15.6 - 16.9) | 16.6 (15.3 - 17.9) | 7.8 (6.8 - 8.9) | 19.1 (15.8 - 22.4) | 20.2 (18.2 - 22.3) | 25.6 (24.3 - 26.8) | 10.6 (9.1 - 12.2) | 7.8 (7.2 - 8.4) | 10.5 (10.1 - 11.0) | 7.7 (6.8 - 8.5) | 10.7 (8.6 - 12.7) | 11.8 (10.7 - 12.9) | 6.8 (5.7 - 7.8) | 4.9 (4.1 - 5.7) | 10.5 (9.2 - 11.9) |
